# Immune Dysregulation after Cardiothoracic Surgery and Incidental Thymectomy: Maintenance of Regulatory T Cells despite Impaired Thymopoiesis

**DOI:** 10.1155/2011/915864

**Published:** 2011-07-06

**Authors:** Nancy J. Halnon, Paige Cooper, Diana Yu Hui Chen, M. Ines Boechat, Christel H. Uittenbogaart

**Affiliations:** ^1^Department of Pediatrics, David Geffen School of Medicine at UCLA, Los Angeles, CA 90095, USA; ^2^Department of Microbiology, Immunology & Molecular Genetics, David Geffen School of Medicine at UCLA, Los Angeles, CA 90095, USA; ^3^Department of Radiology, David Geffen School of Medicine at UCLA, Los Angeles, CA 90095, USA; ^4^UCLA AIDS Institute, David Geffen School of Medicine at UCLA, Los Angeles, CA 90095, USA; ^5^Jonsson Comprehensive Cancer Center, David Geffen School of Medicine at UCLA, Los Angeles, CA 90095, USA

## Abstract

Thymectomy is performed in infants during cardiothoracic surgery leaving many patients with reduced thympopoiesis. An association between immune disorders and regulatory T cells (Treg) after incidental thymectomy has not been investigated. Questionnaires soliciting symptoms of atopic or autoimmune disease and biomarkers were measured in children and adults with congenital heart disease and either reduced or preserved thymopoiesis. Tregs were examined. Atopic or autoimmune-like symptoms and elevated anti-dsDNA antibodies were common after surgery in individuals with low thymopoiesis. Total Treg number and function were maintained but with fewer naïve Treg. TCR spectratypes were similar to other memory T cells. These data suggest that thymectomy does not reduce total Treg number but homeostasis is affected with reduced naïve Treg. Prevalence of autoimmune or atopic symptoms after surgery is not associated with total number or proportion of Tregs but appears to be due to otherwise unknown factors that may include altered Treg homeostasis.

## 1. Introduction

The thymus plays a central role in the generation and maintenance of peripheral T-cell populations including those responsible for modulation of responses to self-antigens [1–3]. Regulation of immune responses is carried out by a specific population of T cells termed regulatory T cells (Treg) [4–6]. These cells carry a distinct phenotype distinguished by surface expression of CD4, elevated expression of CD25 and intracellular expression of the transcription factor Foxp3 required for suppressive activity [4, 7, 8]. In animal models, the thymus has been demonstrated to be necessary for development of Tregs and continued postnatal production is required to prevent autoimmunity [9]. 

While the thymus appears to have an essential role in production of Treg, data suggest that *in vivo* production of these cells can occur as a result of activation of peripheral CD4+ cells by appropriate antigenic stimuli [6, 10–17]. Whether peripheral expansion is adequate to maintain functional Treg populations and prevent autoimmunity in the absence of thymopoiesis in humans is unknown. The impact of incidental thymectomy in infancy on the generation and maintenance of functional Treg is unknown. In many individuals who have undergone incidental thymectomy during cardiothoracic surgical repairs in infancy, thymopoiesis is reduced, often to undetectable levels, compared to those of individuals without surgery [18]. We observed cases of atopic and autoimmune disease among children enrolled in the prior study leading us to speculate that impaired production or maintenance of Treg may have a causative effect. We therefore examined Treg populations in individuals with congenital heart disease to determine the impact of incidental thymectomy on Treg populations, Treg function, and incidence of acquired atopic and autoimmune disease.

## 2. Patients and Methods

### 2.1. Selection of Study Subjects and Clinical Evaluation

Subjects with a history of congenital heart disease presenting for evaluation to the Adult Congenital Heart Disease Clinic or the pediatric cardiology service at UCLA were invited to participate. Subjects were excluded if they had a history of DiGeorge Syndrome or 22q11 chromosomal deletion (by fluorescence in situ hybridization) or recent or current infections. An upper age limit of 35 years was chosen to ensure participation by individuals likely to have undergone cardiothoracic surgery during infancy that may have required sternotomy and resulted in incidental thymectomy. 

59 individuals ranging from 3 days to 35 years in age were enrolled after informed consent was obtained according to a protocol reviewed and approved by the UCLA Medical Institutional Review Board. Medical history was obtained including age, specific congenital cardiac diagnosis, and history of prior surgical procedures. An additional 5 adult subjects with no history of congenital heart disease or cardiothoracic surgery were also recruited and were included with the No Surgery group (*n* = 15). Determination of atopic and autoimmune symptoms was not performed in these subjects. For most subjects, no details are available regarding excision of the thymus during prior surgery. However 39 individuals either had radiological imaging (CT or MRI) of the chest or underwent an initial or repeat surgery for repair or palliation of congenital heart disease allowing direct visualization of the anterior mediastinum and the presence or absence of thymus tissue was noted [19]. Thymus tissue was reported to be “normal” by gross or histologic evaluation in all those in whom records of pathologic examination performed after incidental thymectomy were available.

### 2.2. Quantitation of TREC and Cellular DNA

Genomic DNA was extracted from PBMC and TREC were quantified using real-time polymerase chain reaction (PCR) analysis, using the 5′ nuclease (TaqMan) assay and the Step One Plus PCR System (Applied Biosystems) using forward and reverse primers and probes as previously described [18]. TRECs were quantified against a standard curve of plasmid containing signal joint TREC (kindly provided by D. Douek). Subject classification was accomplished by plotting TREC values for each subject against age.

### 2.3. Measurement of Antidouble Stranded DNA

Antidouble stranded DNA (anti-dsDNA) was measured from plasma specimens by ELISA (Immco Diagnostics, Buffalo, NY). A semiquantitative analysis was performed using calibrators provided and reported as IU/mL of plasma. Subjects less than 6 months of age were excluded from analysis. In addition, plasma samples from 4 subjects were not suitable for analysis and these subjects were excluded.

### 2.4. Identification, Isolation, and Functional Assay of T-Cell Subsets

Flow cytometry was performed on fresh whole blood to determine the percentages of CD3+ CD4+ (CD4+ T cells), CD4+ CD27+ CD45RA+ (naïve CD4+ T cells), and CD4+ CD25^hi^ FOXP3+ T cells (Treg) and proportion of CD4+ CD25^hi^ FOXP3+ Treg that are CD45RA+ or CD45RA− using fluorochrome conjugated antibodies. Cells were acquired on a BD FACSCalibur flow cytometer and the data were analyzed using FLOWJO Software (Treestar Inc. Ashland, OR). Total lymphocyte number was measured in the hospital clinical laboratory using automated methods. For lymphocyte proliferation assay Tregs were isolated from PBMC using the Miltenyi Biotec MACS (Magnetic Cell Sorting) (Miltenyi Biotech, Auburn, CA) system according to manufacturer's instructions by negative selection of CD4+ T cells and removal of CD45RA+ fraction followed by isolation of CD4+ CD45RA− CD25+ (designated Treg) and the CD4+ CD45RA− CD25− (designated CD25−) CD4+ T cell populations. Responder PBMCs were isolated using Ficoll gradient. For lymphocyte proliferation responding PBMC were incubated with either the CD25− CD4+ T-cell populations or Tregs at 3 : 1 and 1 : 1 ratios/well for a total of 3 × 10^5^ cells for each assay. Samples were incubated in RPMI medium containing 10% human AB serum and 200 ng/mL of anti-CD3 in triplicate for 6 days at 37°C. Tritiated thymidine was added on the 6th day and after 20–24 hours thymidine incorporation was measured and reported as stimulation index.

### 2.5. Quantitative Spectratyping for TCR-V*β* Families

Cryopreserved PBMCs were sorted on a FACSAria II (BD Biosciences, San Jose, CA) to isolate the following populations: CD3+/CD4+/CD45RA+ (naïve), CD3+/CD4+/CD45RA−/CD25^low^ (memory), and CD3+/CD4+/CD45RA−/CD25^high^ (Treg) [8]. RNA was isolated using columns (Qiagen Sciences, MD, 20874). cDNA was generated using High Capacity cDNA Reverse Transcription Kit (Applied Biosciences, Foster City, CA) with the addition of an RNase Inhibitor (Invitrogen Corp, Carlsbad, CA). Real-time PCR (IQ5, Bio-Rad, USA) and size distribution analysis were performed for each V*β* family after amplification of 24 V*β* families by TaqMan probe-based real-time PCR using TCR V*β* family-specific forward primer and constant region reverse primer along with labeled probe. The amplified PCR products were then resolved by capillary electrophoresis and the V*β* family specific size peaks were detected based on the dye-labeled reverse primers (3130 Genetic Analyzer, Applied Biosystems, USA). The resulting histograms for each V*β* family were analyzed and the area under the curve of each size-peak was assessed using Peak Scanner Software v1.0 (Applied Biosystems). Relative size for each peak was reported after calculating the ratio of the signal intensity for the sample peak to reference standard. CDR3 length distribution for naïve CD4+ T cells for 3 healthy controls were analyzed and average area determined for each peak of all 24 V*β* families was calculated. The sum of absolute error from this “reference profile” for peaks in all families was calculated for the entire repertoire as previously described [32]. The sum of error per V*β* family was calculated to derive the percent error from the reference distribution. The value obtained for Tregs was compared to naïve and memory cells in each group of subjects using Wilcoxon rank sum tests.

### 2.6. Statistical Analysis

TREC number and T-cell subsets were treated as continuous variables. Anti-dsDNA antibody values were subjected to square root transformation prior to analysis. Individual differences between groups were analyzed using the Wilcoxon rank-sum test. Associations between age and naïve CD4+ or Treg subpopulations were examined using linear regression. The prevalence of symptoms inquired about in each organ system was compared between subject groups using Fisher's Exact Test. Unadjusted odds ratio was determined for the presence or absence of any symptoms (not organ specific) for each group compared to individuals with no surgery with significance determined using the Mantel-Haenszel statistic. Multivariable logistic regression was performed to determine the age adjusted odds ratio with age as a covariate in subjects with CHD but no history of surgery as the reference group.

## 3. Results

### 3.1. Identification of Subjects with Impaired Thymopoiesis

**Figure 1:**
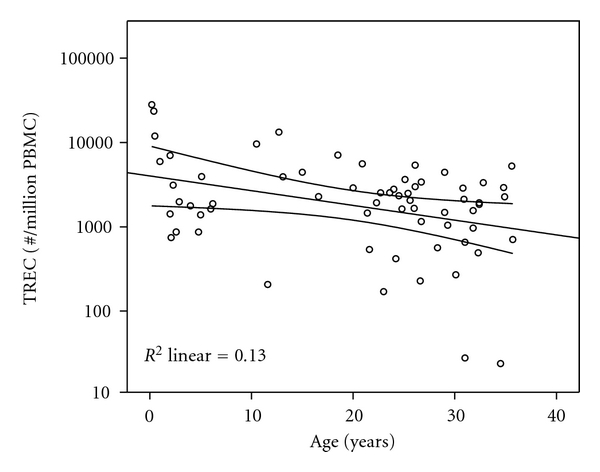
TREC values versus age for subject classification. Age adjusted mean and 99% confidence interval for the mean are displayed. Subjects with TREC values falling below the 99% confidence interval are included in the “low TREC” group.

Subjects with congenital heart disease aged 2 months to 35 years were examined between 2 months and 34.7 years after surgery if any was performed. Normal healthy subjects used for comparison were aged 20 to 29 years old. T-cell recombination excision circles (TRECs) provide a reliable estimate of thymopoiesis [21, 22] and were used to identify subjects with minimal levels of thymopoiesis after cardiothoracic surgery for analysis. TREC values for all groups including healthy adult subjects and for those with CHD showed an age-related decline (Figure 1) and lower values within the subset of subjects with a history of surgery. Subjects with the lowest quartile of TREC values were included in the “Prior Surgery-Low TREC” group (*n* = 17) if TREC number fell below the 99% confidence interval (CI) of the mean for TREC adjusted for age (Figure 1). The remaining subjects were grouped according to surgical history into “No Surgery” or “Prior Surgery High TREC” groups as appropriate. TREC values were not different for subjects with CHD and no surgery (*n* = 10) and healthy controls (*n* = 5) (*P* = .095, ANCOVA); therefore all subjects with no prior surgery are included in the “No Surgery” group (*n* = 15). Subject demographics are displayed in Table 1.

As expected, all subjects within the “Low TREC” group have a history of prior surgery and, for those who were evaluated most had no residual thymus tissue (9 without versus 2 subjects with residual thymus tissue). Compared to other subjects who underwent surgery, those with low TREC more frequently had surgery during infancy (compared to older age) and a larger proportion underwent two surgeries or more (compared to a single surgery). Age at 1st surgery was lower for these subjects; time since 1st surgery, age at last surgery (including those who underwent more than 1 prior procedure), and time since last surgery were similar for those with prior surgery and low TREC versus high TREC. We believe that under our classification scheme we have identified subjects who have significant long-term disruption to normal de novo T-cell production via thymopoiesis [23–25]. As seen in previous studies, the absolute number and percentage of CD4+ T cells was reduced in subjects with prior surgery and low TREC (data not shown).

### 3.2. Presence or Absence of Symptoms Is Associated with Prior Cardiothoracic Surgery

Subjects with CHD were asked to self-report chronic, frequent, recurrent, or long-standing symptoms including seasonal or respiratory allergic symptoms; asthma; chronic skin rashes; joint pain, redness, or swelling; chronic or recurrent abdominal pain or diarrhea; diabetes; or thyroid disorders. The age of onset was also elicited if subjects could recall. To ensure better homogeneity among subjects, normal volunteers without congenital heart disease were excluded from this analysis. One subject did not complete the questionnaire. Therefore, analysis included the remaining 58 subjects with CHD. We hypothesized that older subjects would have a cumulative experience including more opportunity to develop chronic symptoms and so analysis was repeated including age as a covariate in logistic regression and reported as age-adjusted OR (Table 2). Positive responses were frequent in those having a history of prior surgery with 63% of subjects reporting the presence of at least one chronic or longstanding symptom (Table 2). Subjects with low levels of thymopoiesis were affected more frequently than those with CHD but no surgery even after adjustment for age. Of note, skin was most frequently affected organ, particularly in those with low thymopoiesis with 7/17 (41%) complaining of symptoms (Table 3), significantly more often than in those without surgery: no subject with CHD who had not undergone surgery reported any skin symptoms. Among the 14 subjects reporting skin symptoms, eczema was the single most frequent diagnosis in 5 subjects (2 with high TREC and 3 with low TREC). One subject reported contact allergy. Six reported the presence of rash including redness with or without itching or scaling but with no definite or specific diagnosis given. Data from population-based studies indicate that the incidence of skin symptoms in the subjects after surgery is similar to that reported for atopic and eczematous skin conditions in large population-based questionnaire studies [26]. While four subjects in the low TREC group and two in the high TREC group reported having both skin and joint symptoms, no subjects reported a diagnosis of systemic lupus erythematosis (SLE).

Odds of having symptoms were higher in the 19 subjects lacking thymus tissue (after surgical procedures) to the 20 subjects in whom thymus was present (6 without and 14 with prior surgery) (OR 4.47; 95% CI 1.19–18.4; *P* = .027). These data suggest that thymectomy and level of thymopoiesis are associated with the development of autoimmune or atopic symptoms. 

### 3.3. Anti-dsDNA Antibody Titers Are Increased in Patients with Low TREC

**Figure 2:**
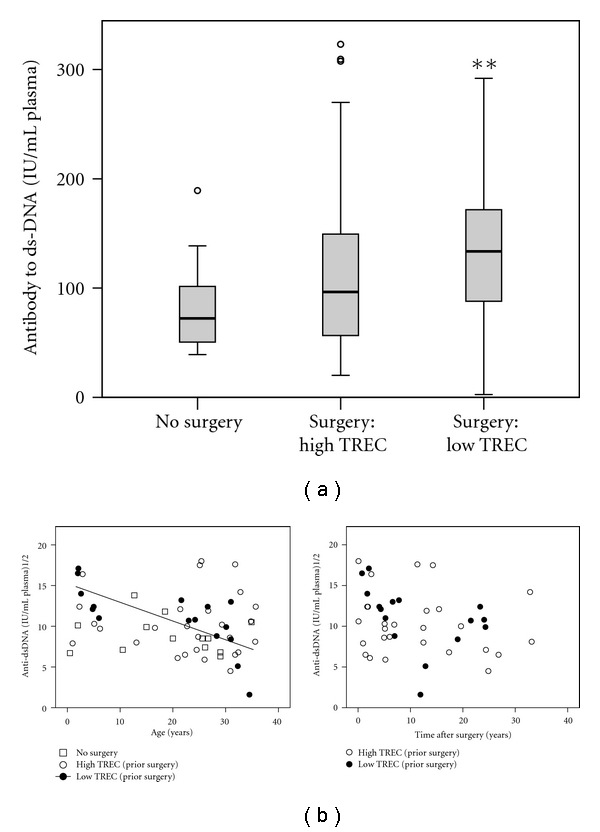
Anti-dsDNA. (a) Anti-dsDNA antibody levels are displayed. Levels were compared between groups using the Wilcoxon rank sum test (**P = .03 versus No Surgery; 2-tailed asymptotic). (b) Square root transformed anti-dsDNA levels are plotted by age (left panel) and time after last surgery (right panel). Associations were examined using linear regression and the significant trendline for the “low TREC” group is displayed (*P* = .002, adj R^2^ = .477). For the other groups there is no significant association between anti-dsDNA level and age or time since last surgery.

Multiple case reports have suggested an association of thymectomy to later development of SLE [27–29] though these reports include patients with myasthenia gravis, an autoimmune disorder. Among immune biomarkers, anti-dsDNA has been associated with autoimmune disease, particularly SLE. In order to assess whether thymectomy itself correlated with levels of this biomarker, we measured plasma concentration of antibody to double stranded DNA (dsDNA) in subjects older than 6 months old (to avoid detection of maternal transplacentally acquired antibody) with high and low levels of thymopoiesis after surgery and healthy controls. Antibody levels in subjects with low thymopoiesis were increased compared to subjects with no surgery (median 133.6 IU/mL versus 96.5 IU/mL; *P* = .03) (Figure 2(a)). To further elucidate this relationship, we examined antibody levels by age. There was no association of antibody level with age in subjects with no surgery or those with a history of prior surgery and higher TREC levels. However, there is a negative correlation with age in subjects with low TREC (Figure 2(b)). While there is no statistically significant relationship between time after surgical procedure and anti-dsDNA, there is a tendency for levels to decrease over time possibly accounting for the relationship seen with age. Thus it appears that production of anti-dsDNA may be initiated near the time of surgical procedures and levels diminish with time over the course of years. Interestingly, level of anti-dsDNA were not associated with the presence or absence of symptoms nor associated with presence or absence of residual thymus tissue.

### 3.4. Total Treg Are Not Dependent on Thymopoiesis for Maintenance

**Figure 3:**
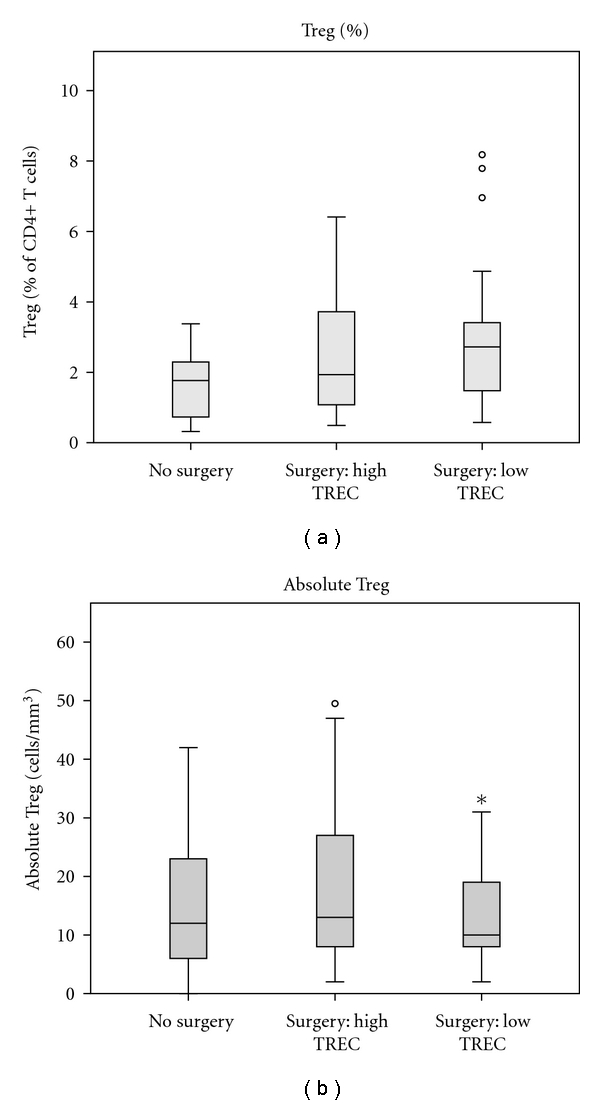
Quantification of Tregs. Percent and absolute number of CD4+/CD25+/Foxp3+ Tregs are displayed. Groups were compared using Wilcoxon rank sum with no differences found based on level of thymopoiesis and history of surgery.

We used 4-color flow cytometry to quantify Treg (see Figure S1 in the supplementary Material available online at doi: 10.11551 201110915864). Treg were defined as CD4+/CD25+/Foxp3+ T cells. We quantified both CD45RA+ and CD45RA− Treg populations. In all subjects including those with reduced thymopoiesis, the highest expression of CD25 was present on the CD45RA− Treg population [7, 8]. There was no relationship between age and total Treg number or frequency (percent of CD4+ T cells) among subjects in any group (data not shown). Neither absolute numbers of CD4+ Treg nor their proportion among all CD4 T cells differed between groups regardless of history of surgery or level of thymopoiesis and TREC number (Figure 3). 

**Figure 4:**
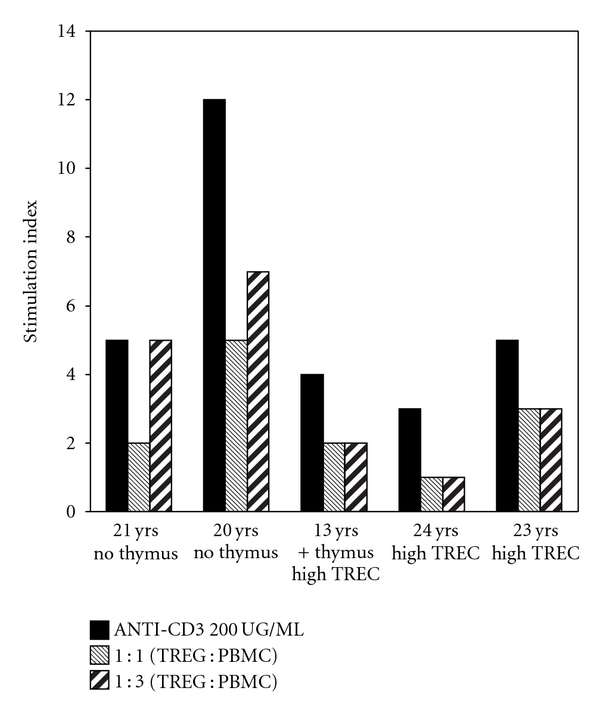
Lymphocyte proliferation. PBMC from 5 subjects were stimulated with 200 ng/mL anti-CD3 antibody. Stimulation index (representing the mean of triplicate wells for each subject and each condition) in the presence of no added Tregs (black bars). Proliferation was suppressed by addition of Treg at a ratio 1 : 1 Treg to PBMC (grey bars), or at a ratio of 1 : 3 Treg to PBMC (hatched bars) in each subject.

Functional capacity was determined for Tregs in a subset of 5 adolescent and adult subjects (2 with sternotomy during infancy and no residual thymus, 1 with surgery during infancy and residual thymus and high thymopoiesis, and 2 with surgery after infancy and high thymopoiesis, presence of residual thymus tissue unknown). Proliferation of PBMC was measured after stimulation with anti-CD3 antibody without or with the addition of CD25+/CD4+ Treg in 2 concentrations 1 : 1 or 1 : 3 (Treg: PBMC). In each subject, Tregs had the ability to suppress proliferation of PBMC *in vitro* in a dose-dependent manner regardless of history of sternotomy or thymectomy (Figure 4). Proportion or absolute Treg was no different between subjects with or without symptoms after adjusting for age. Similarly, level of anti-dsDNA was not associated with Treg number or percentage. Taken together, these data suggest that while surgery is associated with symptoms commonly found in autoimmune or atopic disease, alterations in total Treg number or proportion are not part of the causative mechanism.

### 3.5. Naïve Treg Populations Are Affected by Age and Thymopoiesis

**Figure 5:**
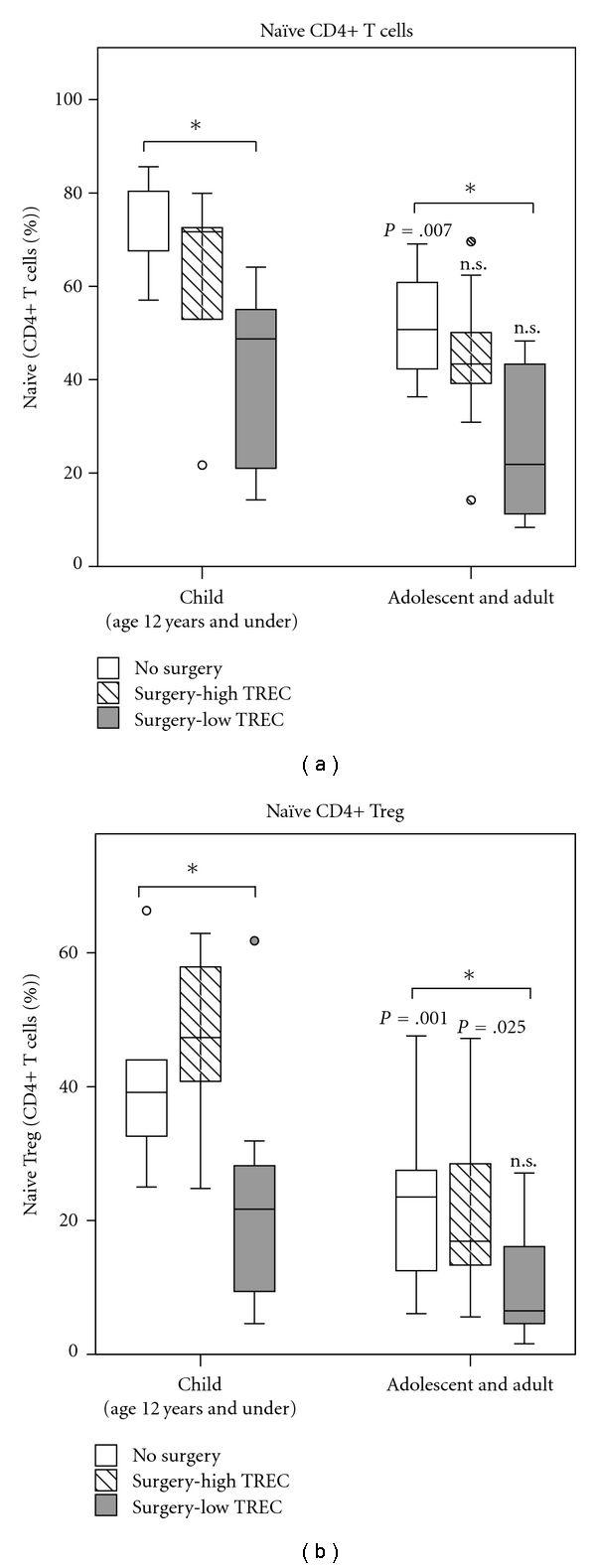
Proportion of naïve CD4+ and Tregs. Proportion of CD4+ T cells and Tregs that are naïve (CD27+CD45RA+ or CD45RA+ for Treg) were examined by age group. In subjects with no surgery, proportion of CD4+ T cells and CD4+ Tregs that are naïve decrease between childhood and adulthood (*P* = .007, *P* = .025; Mann-Whitney test). In subjects with low TREC median proportion of naïve cells is reduced compared with no Surgery at each age (*P < .03), but there is no difference between children and adults (*P* = n.s. for both; Mann-Whitney test).

In total CD4+ T cells, homeostasis of naïve populations is at least in part due to thymopoiesis. Naïve (CD45RA+) as a proportion of Treg is highest in infancy and falls rapidly with age [30]. In our study we investigated the proportion of naïve Treg and naïve (CD27+/CD45RA+) CD4+ T cells by age. We find that the proportion of Treg with a naïve phenotype is associated with age and shows a rapid decline after infancy accounting for 10–20% of Treg in adults. In subjects with no prior surgery there is a similar decline with age for both naïve CD4+ T cells and Treg compared with subjects with prior surgery and low TREC in whom the proportion of naïve cells is similar in both adults and children (Mann-Whitney test) (Figure 5). While total Treg are maintained in subjects with low TREC, the proportion of naïve (CD45RA+) to memory cells (CD45RA−) within the Treg population is affected by surgery, thymectomy, and level of thymopoiesis. Median proportion of naïve CD4+ and naïve among Tregs are lower in those with low TREC compared to no surgery both during childhood and in adolescents/adults. While this could be due to depletion occurring at the time of surgery, the relationship with age and similarity to other naïve CD4+ T cells would suggest that naïve Treg are normally produced via thymopoiesis. When this process is disturbed, total Treg are maintained by an active homeostatic process which favors production of cells with a nonnaive phenotype. 

### 3.6. TCR Spectratype in CD4+/CD25+/CD45RA− Cells Shows Multiple Expansions

**Figure 6:**
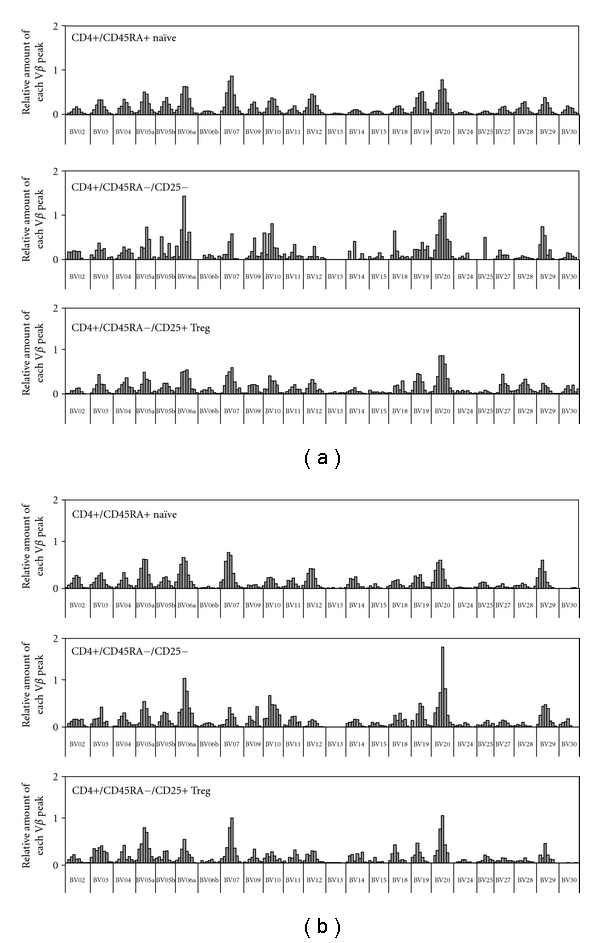
Histogram representations of spectratype for CD4+ T-cell subpopulations. Representative data from 2 subjects is displayed. Distribution of peak lengths for all V*β* families for an 18-year-old with no surgery (a) and a 2-year-old after multiple prior surgeries and low TREC. (b) Relative Vb lengths for naïve (CD45RA+), memory (CD45RA−), or non-naïve Treg (CD45RA−CD25+) CD4+ T cells are displayed.

Patterns of TCR V*β* usage have been examined by others and show unequal usage of different V*β* families in Treg, but largely similar to total CD4+ T cells [31, 32]. We wanted to characterize the TCR repertoire of Treg after thymectomy and to quantify clonal expansions within the V*β* families. TCR spectratype was evaluated in sorted non-naïve Treg (CD4+/CD25+/CD45RA−) and compared to naïve (CD4+/CD25−/CD45RA+) cells and memory (CD4+/CD45RA−) T cells. Therefore we were able to compare a thymus generated (naïve) T-cell population, a peripherally expanded (memory) population, and non-naïve Treg. Naïve CD4+ T cells show Gaussian distributions in most V*β* families, a pattern typically seen in thymus-derived subpopulations. In contrast, spectratyping demonstrated perturbations among TCR lengths in many V*β* families in sorted CD45RA− Treg, indicative of clonal expansions in peripheral T cell compartments (Figures 6(a) and 6(b)). As expected, memory CD4+ T cells show perturbations in TCR spectratype suggestive of multiple clonal expansions in many V*β* families. The extent of perturbation was quantified by assessing the error for each V*β* family from a reference spectratype consisting of the average of 3 naïve CD4+ T-cell specimens obtained from healthy adults [33]. Average error from the reference standard in naïve CD4+ was 7.03 ± 0.59% in healthy adults; 8.20 ± 3.30% for subjects with CHD and no surgery; 11.9 ± 4.31% for subjects with CHD and prior surgery (2 with High TREC and 1 with LOW TREC). In contrast Treg spectratypes contain more error, an average of 7.68 ± .37% (healthy controls); 14.1 ± 1.99% (CHD no surgery); 19.2 ± 8.86% (after surgery) (for all *P* = .028 versus naïve). Memory spectratypes were not statistically different from Treg (*P* = .600, Wilcoxon rank-sum). These data suggest that maintenance of the memory Treg subpopulation comprising the majority of Treg after infancy is not dependent on thymic production, but rather is typical of memory populations and is derived from peripheral homeostatic proliferation or production of induced Treg. 

## 4. Discussion

Many infants requiring cardiothoracic surgery undergo incidental thymectomy. Some of the long-term effects on T-cell production have been described but to date immune dysregulation has not been fully investigated in these individuals [18, 24, 34, 35]. Studies in animal models have provided the basis for a number of hypotheses. In day 3 thymectomy mice, endogenous Treg derived from peripheral replication are not sufficient to fully control autoimmunity [36]. A number of investigators have hypothesized that thymus-derived Treg are distinct from induced Treg derived from activation and proliferation of CD25−CD4+ T cells in the periphery [10, 20, 37]. While both types are identified by similar phenotypic markers (CD4+/CD25+/FoxP3+), they may differ in functional capacity or specificity and the presence of both types may be required to perform synergistically or perform distinct nonoverlapping roles in the maintenance of self-tolerance and control of autoimmunity [37]. 

In this study we found evidence of immune dysregulation among the subjects with the most impairment of thymopoiesis and reduction in number of naïve, thymus-derived CD4+ T cells and naïve CD4+ Treg. This would support the hypothesis that thymus-derived Treg are a distinct population, maintained separately from peripherally expanded induced Treg with different functional capacity. A recent study suggests that in addition to production of functional Treg, the thymus may also be an important contributor to committed CD4+CD25+Foxp3− Treg precursors, providing a further mechanism for disruption of homeostasis in athymic individuals [38]. Potentially, further investigation could better reveal the thymic versus peripheral origin of Treg possibly based on compartmentalization of PD-1 or expression of helios [39, 40]. In addition, subtle disturbance of positive or negative selection that may occur in these subjects could not be identified clinically and therefore, potential effects of selection cannot be ruled out. A number of groups have suggested that a CD45RA+ phenotype may be associated specifically with thymus-derived Treg because the proportion of CD45 RA+ to CD45RA− Treg varies with age at a similar rate to the decline in thymopoiesis [30, 41]. Furthermore, recent data has shown that Treg that exit the thymus are exclusively CD45RA+ (Douaisi et al., manuscript submitted). In the current study, we see an age-related decline in CD45RA+ Treg, and this effect is exaggerated by sternotomy, thymectomy, and level of thymopoiesis similar to the age-related decline in total naïve (non-Treg) CD4+ T cells in these subjects. Our data tend to confirm that ongoing thymopoiesis is not a requirement for total Treg homeostasis. However, homeostasis of naïve Treg is affected by surgery and thymopoiesis in a pattern confirming that non-naïve (CD45RA−) Treg may be produced in the periphery by proliferation of existing Treg or other CD4+ T cells. Naïve Tregs appear to be thymus derived resulting in reduction of this population in our subjects with low TREC and reduced thymopoiesis.

There may be other possibilities to explain the increased frequency of chronic symptoms seen in our subjects with prior surgery and reduced thymopoiesis as compared to subjects with CHD but no history of surgery. Age differences alone do not seem to account for this difference as the group with no surgery shows a similar age distribution to those with low TREC. In addition the most frequently reported symptoms are those suggestive of eczema and atopic dermatitis. Recent literature provides a wide range for the worldwide prevalence of eczema and atopic dermatitis among children, from less than 1% to more than 20% in childhood during the peak ages [26]. Prevalence is likely to be lower in adults but may vary based on ethnicity, nationality, and occupational exposures [42–46]. Allergic rhinoconjunctivitis is common in children as well with a prevalence of less than 2% to nearly 40% depending on nation surveyed [26]. Given the wide variation reported it would be difficult to conclude from our data that the frequency of symptoms is outside normal ranges, however given the geographically limited cohort, the difference between groups should be given consideration. Eczema and atopic dermatitis tend to occur most frequently in children with onset by one year of age 60% of the time [26, 42]. Similarly we found that onset of symptoms, when they occurred, was frequently during childhood and the young age of some subjects is unlikely to be the primary reason for differences between groups. The epidemiology of this disease has suggested many risk factors including genetic predisposition and occupational exposures which were not extensively evaluated in this study. Consequently, confounding factors have not been adequately addressed and would deserve further study. The number of subjects with unoperated CHD is small and may be inadequately powered to detect differences. However, when analyzed strictly on the basis of presence or absence of thymus tissue, we found that subjects without versus those with thymus tissue report symptoms more frequently suggesting that incidental thymectomy or related factors are important. Recall bias may affect the reporting of symptoms, particularly in subjects who have had surgery and high levels of medical contact compared to those who have had no surgical procedures. However except for an earlier age at first surgery, there are no important demographic differences apparent in those with higher and lower thymopoiesis that could account for group-related differences in recall. It is interesting to note case reports attributing cases of SLE to thymectomy performed for myasthenia gravis, a disease that itself is thought to be immune mediated [27–29]. Cardiothoracic surgery and cardiopulmonary bypass are known to produce short-term immune activation regardless of thymectomy [47–49]. Long-term effects have not been described. Immunologic effects seen in these children and young adults may be multifactorial and related to features of surgery in addition to thymectomy. Further study using more nuanced methods to document autoimmunity would be useful.

## 5. Conclusions

Our data suggest that there are long-term immune effects after cardiothoracic surgeries for repair or palliation of congenital heart disease that have not been described to date. While total Treg number is maintained, homeostasis of this population is affected with unclear affects on autoimmunity. Subjects with the most impairment in thymopoiesis, typically those having multiple surgeries beginning in infancy, correspond to those with elevations in anti-dsDNA antibody and more self-reported symptoms. As well, these subjects show evidence of alterations in Treg homoeostasis that may play a contributory role. The differences we have detected by the crude measures employed argue for further study using more nuanced clinical diagnostic criteria and expanded biomarker measurements. Further study of immune dysregulation in individuals subjected to cardiothoracic surgery, particularly in infancy, is warranted.

## Figures and Tables

**Table 1:** Characteristics of subjects.

	No Surgery	Prior Surgery: high TREC	Prior Surgery: low TREC
	*n* = 15 (10-CHD; 5-healthy adults)	*n* = 32	*n* = 17
Age at evaluation (mean years; range)*	16.47 (0.2–34.9)	22.6 (1.00–35.7)	18.6 (2.00–34.5)
Healthy adults	26.1 (20.0–29.0)		
CHD	12.0 (0.2–34.9) *P* = .02^∧^ versus Healty adults		
Age at 1st surgery		6.6 (0–28.4)	2.5 (0–13.8) *P* = .02^∧^ versus High TREC
Time since 1st surgery		16.0 (0.2–34.7)	16.1 (1.9–34.0)
Age at last surgery		12.2 (0.01–34.71)	1.4 (0.03–24.4)
Time since last surgery		10.4 (0.1–33.1)	11.0 (0.8–24.3)
Number of surgeries (number of subjects)			
0	15 (100%)		
1		16 (50%)	5 (29%)
≥2		16 (50%	12 (71%)
Age at initial surgery (number of subjects)			
<2 years		13 (41%)	12 (71%)
>2 years		19 (59%)	5 (29%)
Residual thymus (present : absent)**	6 : 0	12 : 10	2 : 9

Surgery = midline stemotomy.

*Kruskal-Wallis test = n.s.

**Chi-Square test *P* = .005.

^∧^Mann-Whitney test.

**Table 2:** Symptoms reported by subjects with CHD.

	Positive responses	OR (95% Cl)	Age adjusted OR (95% Cl)
No Surgery	2/10 (20%)	1	1
Any surgery (*n* = 48)	30/48 (63%)	6.67 (1.27–34.9) *P* = .032	4.88 (.877–27.1) *P* = .070
High TREC (*n* = 31)	18/31 (58%)	5.54 (1.01–30.5) *P* = .067	4.09 (.069–24.5) *P* = .122
Low TREC (*n* = 17)	12/17 (71%)	9.60 (1.48–62.1) *P* = .018	8.47 (1.12–64.3) *P* = .039

**Table 3:** Symptoms reported by organ or system (number of subjects).

	Allergic	Gastrointestinal	Respiratory	Joints	Endocrine	Skin
No surgery (*n* = 10 with CHD)	1 (10%)	1 (10%)	0	1 (10%)	0	0
Any surgery (*n* = 48)	13 (27%)	12 (25%)	8 (17%)	13 (27%)	4 (8%)	15 (31%)
High TREC (*n* = 31)	9 (29%)	9 (29%)	3 (10%)	8 (26%)	3 (10%)	7 (23%)
Low TREC (*n* = 17)	4 (24%)	3 (18%)	5 (29%)	5 (29%)	1 (6%)	7 (41%)*

*P = .026 versus No Surgery (Fisher exact test).

## Abbreviations

Treg: Regulatory T-cell
CHD: Congenital heart disease
PCR: Polymerase chain reaction
PBMC: Peripheral blood mononuclear cells
TREC: T cell receptor recombination excision circles
dsDNA: Double-stranded DNA
SLE: Systemic lupus erythematosis.
